# Autism spectrum disorders detection based on multi-task transformer neural network

**DOI:** 10.1186/s12868-024-00870-3

**Published:** 2024-06-13

**Authors:** Le Gao, Zhimin Wang, Yun Long, Xin Zhang, Hexing Su, Yong Yu, Jin Hong

**Affiliations:** 1 School of Computer Engineering, Guangzhou Huali College, Guangzhou, 511325 China; 2 Faculty of Intelligent Manufacturing, Wuyi University, Jiangmen, 529000 China; 3 State Key Laboratory of Public Big Data, Guizhou University, Guizhou, 550025 China; 4 School of Computer Science, Shaanxi Normal University, Xi’an, 710062 China; 5 School of Information Engineering, Nanchang University, Nanchang, 330031, China

**Keywords:** Autism Spectrum Disorders, Artificial intelligence, Biological information, Multi-task learning, Transformer network

## Abstract

Autism Spectrum Disorders (ASD) are neurodevelopmental disorders that cause people difficulties in social interaction and communication. Identifying ASD patients based on resting-state functional magnetic resonance imaging (rs-fMRI) data is a promising diagnostic tool, but challenging due to the complex and unclear etiology of autism. And it is difficult to effectively identify ASD patients with a single data source (single task). Therefore, to address this challenge, we propose a novel multi-task learning framework for ASD identification based on rs-fMRI data, which can leverage useful information from multiple related tasks to improve the generalization performance of the model. Meanwhile, we adopt an attention mechanism to extract ASD-related features from each rs-fMRI dataset, which can enhance the feature representation and interpretability of the model. The results show that our method outperforms state-of-the-art methods in terms of accuracy, sensitivity and specificity. This work provides a new perspective and solution for ASD identification based on rs-fMRI data using multi-task learning. It also demonstrates the potential and value of machine learning for advancing neuroscience research and clinical practice.

## Introduction

ASD (Autism Spectrum Disorders) is a heterogeneous condition that affects communication, behavior, and social interactions in various ways and degrees [1]. According to the latest Diagnostic and Statistical Manual of Mental Disorders (DSM-5), ASD encompasses a spectrum of disorders that were previously diagnosed separately, such as autism, Asperger’s syndrome, and other pervasive developmental disorders. The global prevalence of ASD has increased dramatically over the years, reaching 1 in 59 children in the United States in 2014 [2]. ASD poses a major public health challenge, as it impacts not only the individuals with ASD, but also their families and society [3]. Early diagnosis and intervention are crucial for improving the outcomes and reducing the costs of ASD [4], but the current standard diagnosis relies on subjective and time-consuming assessments by multidisciplinary teams using standardized tools [5]. These assessments require highly specialized knowledge and experience from the evaluators, and are often inaccessible or unavailable to many patients [6]. Therefore, there is an urgent need for objective and efficient diagnostic methods based on biological markers.

With the rapid development of AI (Artificial Intelligence) technology, machine learning as a subfield of AI, it has largely enhanced the role of computational methods in neuroscience [7]. Machine learning has been successfully applied in Alzheimer’s disease, mild cognitive impairment [8, 9], temporal lobe epilepsy, schizophrenia, Parkinson’s [10], dementia [11, 12], ADHD [13, 14], ASD [15, 16] and major depressive disorder [17]. In particular, the identification of ASD has made great progress and a series of effective methods have been developed [18]. These methods can be briefly divided into two categories as follows: (1) Based on traditional machine learning methods, it models ASD data as a binary classification problem using traditional machine learning techniques. Crippa et al. [19] used support vector machine (SVM) algorithm to segment ASD patient samples and normal controls (NC) samples by fitting a hyperplane. Rane et al. [20] used logistic regression method to predict ASD diagnosis by transforming fMRI data into probabilities of specific binary values through linear operations. Abbas et al. [21] used an integrated learning approach to construct an ASD screening tool by combining a parent questionnaire-based classifier and a behavioral video-based classifier; (2) Based on the deep learning approach, it uses deep neural networks to extract hidden features in ASD data for ASD identification. Heinsfeld et al. [22] used autoencoders to downscale rs-fMRI data and then used deep neural networks for ASD prediction. Alsaade et al. [23] performed prediction of ASD disease by constructing a functional brain connectivity matrix and projecting it to a deep feature space. Pavăl [24] used convolutional neural networks for facial abnormality identification in ASD patients.

**Fig. 1:**
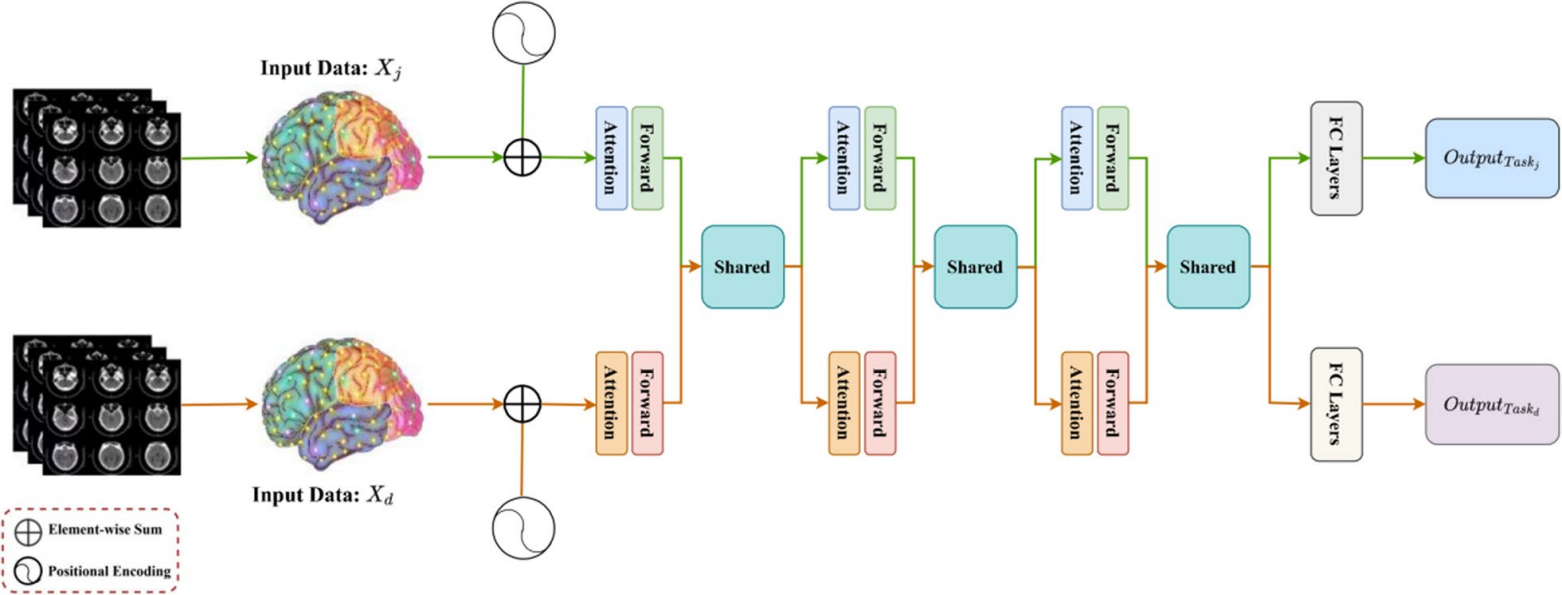
Multitasking transformer framework diagram

Despite the success of these methods, the identification of ASD remains a challenge due to the complex causes of autism formation and unclear pathogenesis [25, 26]. Moreover, most existing methods are based on single-task learning, which ignores the potential correlations and complementarities among different ASD recognition tasks [27, 28]. To address these issues, we propose a novel multi-task learning framework for ASD identification based on resting-state functional magnetic resonance imaging (rs-fMRI) data. Figure 1 is the multitasking transformer framework diagram. Rs-fMRI is increasingly used to study neural connectivity and identify biomarkers of psychiatric disorders. It performs imaging based on blood oxygen level-dependent (BOLD) signal changes in brain regions in a non-invasive manner. Thus rs-fMRI-based ASD identification can provide more accurate, stable and interpretable predictions.

The main contributions and novelties of our work are as follows:

This paper proposes a novel multi-task learning framework for ASD identification based on rs-fMRI data, which can leverage useful information from multiple related tasks to improve the generalization performance of the model. We introduce a temporal encoding module to encode the rs-fMRI data, which can capture the sequential information embedded in the temporal nodes. Meanwhile, we adopt an attention mechanism to extract ASD-related features from each rs-fMRI dataset, which can enhance the feature representation and interpretability of the model.

We design a feature sharing module to share the ASD features learned from each dataset, which can exploit the correlations and complementarities among different tasks.

We conduct extensive experiments on two public rs-fMRI datasets to evaluate the effectiveness of our proposed method. The results show that our method outperforms state-of-the-art methods in terms of accuracy, sensitivity and specificity. This work provides a new perspective and solution for ASD identification based on rs-fMRI data using multi-task learning. It also demonstrates the potential and value of machine learning for advancing neuroscience research and clinical practice.

## Materials and methods

### Materials

In the present study, we used rs-fMRI data from the Autism Imaging Data Exchange (ABIDE). Due to the limited number of subjects at the site, we selected 2 different sites (number of subjects > 100) from a large number of sites, including UM and NYU. Also, data and detailed information are available at https://fcon_1000.projects.nitrc.org/indi/abide/, where Table 1 shows the demographic information of the subjects aggregated.

**Table 1:** Demographic information of subjects

Site	ASD		NC		Total
	Age Avg(SD)	Count	Age Avg(SD)	Count	
NYU	14.76(7.12)	M 64, F 10	15.75(6.18)	M 72, F 26	172
UM	13.71(2.37)	M 38, F 9	14.84(3.62)	M 55, F 18	120

#### Data preprocessing

There is no consensus on the best methods for preprocessing resting state fMRI data. Rather than being prescriptive and favoring a single processing strategy, we have preprocessed the data using Connectome Computation System (CCS), Configurable Pipeline for the Analysis of Connectomes (CPAC), Data Processing Assistant for Resting-State fMRI (DPARSF), Neuroimaging Analysis Kit (NIAK), each of which was implemented using the chosen parameters and settings of the pipeline developers.

The preprocessing steps implemented by the different pipelines are very similar. The largest changes are for the specific algorithms used for each step, their software implementations, and the parameters used. The following sections outline the different preprocessing steps and their differences in the pipeline.

#### Basic processing

**Table:** 

Step	CCS	C-PAC	DPARSF	NIAK
Drop first “N” volumes	4	0	4	0
Slice timing correction	Yes	Yes	Yes	No
Motion realignment	Yes	Yes	Yes	Yes
Intensity normalization	4D Global mean = 1000	4D Global mean = 1000	No	Non-uniformity correction using median volume

### Nuisance signal removal

Each pipeline implemented some form of nuisance variable regression to clean confounding variation due to physiological processes (heart beat and respiration), head motion, and low frequency scanner drifts, from the fMRI signal.

**Table:** 

Regressor	CCS	C-PAC	DPARSF	NIAK
Motion	24-param	24-param	24-param	Scrubbing and 1st principal component of 6 motion parameters and their squares
Tissue signals	Mean WM and CSF signals	CompCor (5 PCs)	Mean WM and CSF signals	Mean WM and CSF signals
Motion realignment	Yes	Yes	Yes	Yes
Low-frequency drifts	Linear and quadratic trends	Linear and quadratic trends	Linear and quadratic trends	Discrete cosine basis with a 0.01 Hz high-pass cut-off

#### Processing strategies

Each pipeline was used to calculate four different preprocessing strategies:

**Table:** 

Strategy	Band-pass filtering	Global signal regression
filt_global	Yes	Yes
filt_noglobal	Yes	No
nofilt_global	No	Yes
nofilt_noglobal	No	No

For strategies that include global signal correction, the global mean signal was included with nuisance variable regression. Band-pass filtering (0.01–0.1 Hz) was applied after nuisance variable regression.

#### Registration

A transform from original to template (MNI152) space was calculated for each dataset from a combination of functional-to-anatomical and anatomical-to-template transforms. The anatomical-to-template transforms were calculated using a two step procedure that involves (one or more) linear transform that is later refined with a very high dimensional non-linear transform. When data are written into template space (typically after the calculation of derivatives, except for NIAK) all transforms are used simultaneously to avoid multiple interpolations.

**Table:** 

Registration	CCS	C-PAC	DPARSF	NIAK
Functional to Anatomical	Boundary-based rigid body (BBR)	Boundary-based rigid body (BBR)	Rigid body	Rigid body
Anatomical to Standard	FLIRT + FNIRT	ANTs	DARTEL	CIVET

### Methods

In this section, we design the multitask Transformer framework to improve the ASD prediction performance by sharing the knowledge learned from multiple tasks. Specifically, Section“Problem definition” formally defines the problem. Section“Location coding” describes how to encode positions according to the order of time nodes. Section“Attention module” defines the way the attention mechanism in the Transformer captures useful features. Section“Feature sharing” describes the process of feature sharing among different tasks. Section“Objective function” defines the objective function for Optimization of the objective function.

#### Problem definition

In this section, we describe the proposed multi-task Transformer learning framework. Suppose we have ${\text{D}}$ tasks and the rs-fmri dataset as follows (1), An instance as follows (2) in ${{\text{X}}}_{{\text{d}}}$ contains ${\text{T}}$ time nodes and ${\text{N}}$ brain regions, and the corresponding label ${{\text{y}}}_{{\text{d}}}\in \{\mathrm{0,1}\}$ is a binary classification task, In the experiment, label 1 represents illness, label 0 represents no disease, and the label and label of both tasks have the same significance. We further assume that there are ${\text{D}}$ different Transformer networks, and each Transformer network consists of L-layer feedforward networks, where the lth layer network extracts the features of task d through ${{\text{f}}}_{{\text{\;d}}}^{{\text{\;l}}}\in {\mathbb{R}}^{{\text{T}}\times {\text{N}}}$. Specifically, our goal is to improve the generalization performance of task d by sharing features learned from other tasks as follows (3).

1 $${{\text{D}}=\{{{\text{X}}}_{{\text{d}}},{{\text{Y}}}_{{\text{d}}}\}}_{{\text{d}}=1}^{{\text{D}}}$$

2 $${\{{{\text{T}}}_{{\text{d}}}(\cdot )\}}_{{\text{d}}=1}^{{\text{D}}}$$

3 $${\{{{\text{f}\;}}_{{\text{j}}}^{{\text{l}}}\}}_{{\text{l}}=1}^{{\text{L}}},\forall {\text{j}}\ne {\text{d}}$$

#### Location coding

Temporal order information in time series data helps to improve model prediction accuracy [29]. To take full advantage of the sequential information embedded in the time nodes in the rs-fMRI data, we inject information about the position in the time node sequence for each input data. Specifically, we obtain a position-encoded PE with the same dimensionality as ${{\text{x}}}_{{\text{d}}}$ using the sine and cosine function, which is calculated as follows (4, 5).

4 $${{\text{PE}}}_{(\mathrm{t }, 2{\text{n}})}={\text{sin}}({\text{t}}/{10000}^{2{\text{n}}/{\text{N}}})$$

5 $${{\text{PE}}}_{(\mathrm{t }, 2{\text{n}}+1)}={\text{cos}}({\text{t}}/{10000}^{2{\text{n}}/{\text{N}}}$$

where t denotes the position of the time node in ${\text{T}}$ time nodes, $2{\text{n}}$ denotes the brain region of even number, and $2{\text{n}}+1$ denotes the brain region of base number. In our model, ${{\text{x}}}_{{\text{d}}}$ represents the input features of the task d. It is a two-dimensional matrix, where each row corresponds to a time node and each column corresponds to a brain region. Thus, the dimension of ${{\text{x}}}_{{\text{d}}}$ is (T, R), where T is the number of time nodes and R is the number of brain regions. Then, the location information embedding, which we implement by summing the location encoding ${\text{PE}}$ and ${{\text{x}}}_{{\text{d}}}$, is calculated as follows (6).

6 $${\widetilde{{\text{x}}}}_{{\text{d}}}={\text{PE}}+{{\text{x}}}_{{\text{d}}}$$

#### Attention module

**Fig. 2:**
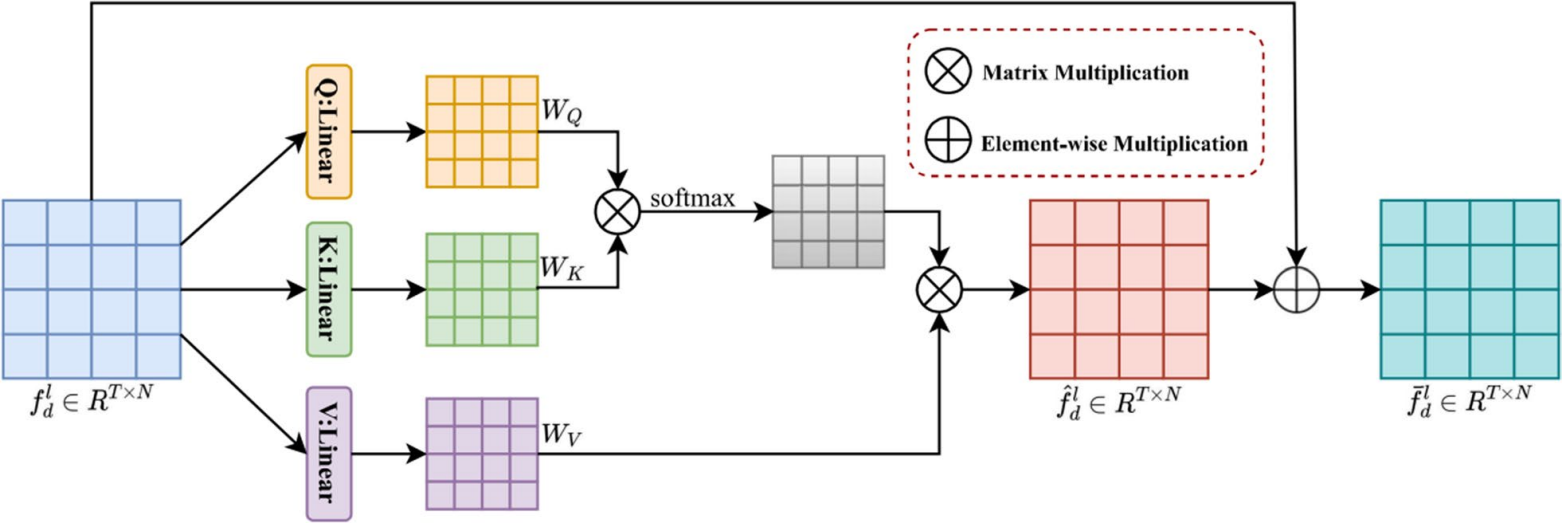
Attention module

The Transformer network consists of several attention modules to improve the ASD prediction performance, as shown in Fig. 2. In our model, Q, K, and V represent the query (Query), the Key (Key), and the value (Value), respectively. These manipulations are central parts of the attention mechanism and are used to compute correlations between input features. ${{\text{f}}}_{{\text{\;d}}}^{{\text{\;l}}}$ represents the l-layer feature of the task d. These features are extracted through feed-forward networks and attention modules and can capture important information about the input data. Our goal is to improve the generalization performance of task d by sharing features learned from other tasks.

Specifically, the features of ${{\text{f}}}_{{\text{\;d}}}^{{\text{\;l}}}$ are first extracted by three different linear operations of Q, K and V, and the number of channels is reduced to half of the original one to reduce the computational effort, as follows (7–9).

7 $${{\text{W}}}_{{\text{Q}}}={\text{Q}}({{\text{f}}}_{{\text{\;d}}}^{{\text{\;l}}})$$

8 $${{\text{W}}}_{{\text{K}}}={\text{K}}({{\text{f}}}_{{\text{\;d}}}^{{\text{\;l}}})$$

9 $${{\text{W}}}_{{\text{V}}}={\text{V}}({{\text{f}}}_{{\text{\;d}}}^{{\text{\;l}}})$$

where ${{\text{W}}}_{{\text{Q}}}\in {\mathbb{R}}^{{\text{T}}\times {\text{N}}}$, ${{\text{W}}}_{{\text{K}}}\in {\mathbb{R}}^{{\text{T}}\times {\text{N}}}$ and ${{\text{W}}}_{{\text{V}}}\in {\mathbb{R}}^{{\text{T}}\times {\text{N}}}$ denote the output feature vectors. Then, we matrix multiply ${{\text{W}}}_{{\text{Q}}}$ ${\text{and}}$ ${{\text{W}}}_{{\text{K}}}$ to calculate the correlation weights between time nodes and score them using softmax operation. Finally, we weight sum the correlation weights and ${{\text{W}}}_{{\text{V}}}$ to obtain the attention feature vector, which is calculated as follows (10).

10 $${\widehat{{\text{f}}}}_{{\text{\;d}}}^{{\text{\;l}}}={\text{softmax}}({{\text{W}}}_{{\text{Q}}}{{{\text{W}}}_{{\text{K}}}}^{{\text{T}}}){{\text{W}}}_{{\text{V}}}$$

where ${\overline{{\text{f}}} }_{{\text{\;d}}}^{{\text{\;l}}}\in {\mathbb{R}}^{{\text{T}}\times {\text{N}}}$ denotes the attention feature vector. Finally, we fuse the attentional feature vector ${\widehat{{\text{f}}}}_{{\text{d}}}^{{\text{l}}}$ and the feature ${{\text{f}}}_{{\text{d}}}^{{\text{l}}}$, which aims to compensate for the information lost when the attentional mechanism captures the features, calculated as follows (11).

11 $${\overline{{\text{f}}} }_{{\text{\;d}}}^{{\text{\;l}}}={\widehat{{\text{f}}}}_{{\text{\;d}}}^{{\text{\;l}}}+{{\text{f}}}_{{\text{\;d}}}^{{\text{\;l}}}$$

where ${\overline{{\text{f}}} }_{{\text{\;d}}}^{{\text{\;l}}}\in {\mathbb{R}}^{{\text{T}}\times {\text{N}}}$ denotes the output fused features. In addition, each layer of the feedforward network consists of an attention module and a Forward network, and the fused features are transformed into the task-specific feature space by a fully connected Forward network, calculated as follows (12).

12 $${\widetilde{{\text{f}}}}_{{\text{\;d}}}^{{\text{\;l}}}={\text{Relu}}({{\text{W}}}_{{\text{f}}}{\overline{{\text{f}}} }_{{\text{\;d}}}^{{\text{\;l}}}+{{\text{b}}}_{{\text{f}}})$$

where ${\widetilde{{\text{f}}}}_{{\text{\;d}}}^{{\text{\;l}}}\in {\mathbb{R}}^{{\text{T}}\times {\text{N}}}$ denotes the output, ${\text{Relu}}(\bullet )$ denotes the activation function, and ${{\text{W}}}_{{\text{f}}}$ and ${{\text{b}}}_{{\text{f}}}$ denote the corresponding parameters.

#### Feature sharing

**Fig. 3:**
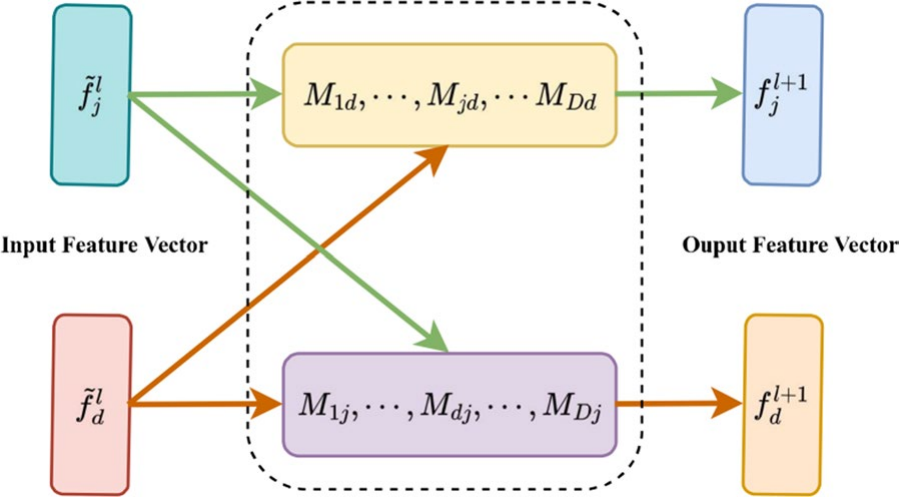
Feature sharing module, in this graph, M represents the activation map for linearly combining the feature vectors of different task networks

To realize the interaction of features between tasks, we build a feature sharing module, as shown in Fig. 3. Each layer of the network defines D learnable activation mappings ${{\text{M}}}_{{\text{D}}}={\{{{\text{M}}}_{{\text{d}}}\}}_{{\text{d}}=1}^{{\text{D}}}$, where ${{\text{M}}}_{{\text{d}}}=\{{{\text{M}}}_{1{\text{d}}},...,{{\text{M}}}_{{\text{Dd}}}\}$. We use ${{\text{M}}}_{{\text{D}}}$ to linearly combine the feature vectors of different task networks and use them as inputs for the next layer of feedforward networks. Specifically, we matrix the activation mapping $${{\text{M}}}_{{\text{D}}}=\left|\begin{array}{ccc}{{\text{M}}}_{11}& ...& {{\text{M}}}_{{\text{D}}1}\\ ...& {{\text{M}}}_{{\text{dd}}}& ...\\ {{\text{M}}}_{1{\text{D}}}& ...& {{\text{M}}}_{{\text{DD}}}\end{array}\right|$$ and use it to linearly combine multiple feature vectors, which are computed as follows (13).

13 $$\left|\begin{array}{c}{{\text{f}}}_{\;1}^{{\text{\;l}}+1}\\ \vdots \\ {{\text{f}}}_{{\text{\;d}}}^{{\text{\;l}}+1}\\ \vdots \\ {{\text{f}}}_{{\text{\;D}}}^{{\text{\;l}}+1}\end{array}\right|=\left|\begin{array}{ccc}{{\text{M}}}_{11}& ...& {{\text{M}}}_{{\text{D}}1}\\ ...& {{\text{M}}}_{{\text{dd}}}& ...\\ {{\text{M}}}_{1{\text{D}}}& ...& {{\text{M}}}_{{\text{DD}}}\end{array}\right|\left|\begin{array}{c}{\widetilde{{\text{f}}}}_{\;1}^{{\text{\;l}}}\\ \vdots \\ {\widetilde{{\text{f}}}}_{{\text{\;d}}}^{{\text{\;l}}}\\ \vdots \\ {\widetilde{{\text{f}}}}_{{\text{\;D}}}^{{\text{\;l}}}\end{array}\right|$$

where ${{\text{f}}}_{{\text{d}}}^{{\text{l}}+1}$ denotes the output of the l + 1-layer network. We can identify specific layer tasks by setting ${{\text{M}}}_{{\text{i}},{\text{k}}}$, ${\text{i}}<{\text{D}},{\text{k}}<{\text{D}}$ to zero, or share more features by assigning them higher values.

#### Objective function

Feedforward networks do not change the dimensionality of the feature vectors; however, high-dimensional and high-noise data have a negative impact on the prediction performance. To solve this problem, we reduce the dimensionality of the feature vector ${{\text{f}}}_{{\text{d}}}^{{\text{L}}}\in {\mathbb{R}}^{{\text{T}}\times {\text{N}}}$ by FC Layers and perform the prediction. FC Layers consists of three layers of fully connected operations, the first two layers are used to reduce the dimensionality and the last layer gets the prediction output, which is calculated as follows (14):

14 $${\widehat{{\text{y}}}}_{{\text{d}}}={\text{softmax}}({{\text{W}}}_{3}({\text{Relu}}({{\text{W}}}_{2}({\text{Relu}}({{\text{W}}}_{1}{{\text{f}}}_{{\text{d}}}^{{\text{L}}}+{{\text{b}}}_{1}))+{{\text{b}}}_{2}))+{{\text{b}}}_{3})$$

where ${\widehat{{\text{y}}}}_{{\text{d}}}$ denotes the output, and ${{\text{W}}}_{{\text{i}}=\mathrm{1,2},3}$ and ${{\text{b}}}_{{\text{i}}=,\mathrm{1,2},3}$ denote the corresponding parameters. Then, we use the binary cross-entropy as the loss and the objective function is calculated as follows (15).

15 $${\mathcal{L}} = \sum\limits_{{{\text{d}} = 1}}^{{\text{D}}} {\left[ {\sum\nolimits_{{\frac{{{\text{x}}_{{\text{d}}} \in {\text{X}}_{{\text{d}}} }}{{{\text{y}}_{{\text{d}}} \in {\text{Y}}_{{\text{d}}} }}}} { - {\text{y}}_{{\text{d}}} {\text{log}}{\mkern 1mu} \widehat{{\text{y}}}_{{\text{d}}} } } \right]}$$

## Results and discussion

In this section, we conduct extensive experiments to verify the effectiveness of our approach. Specifically, Section“Experimental setup” describes the experimental setting and setup. Section“Evaluation metrics” gives the evaluation metrics to evaluate the experimental results. Section“Experimental results and discussion” presents the comparison of our method with the current popular methods on two ASD datasets and the analysis of the experimental results.

### Experimental setup

The experiments are programmed and implemented as follows: PyTorch 1.9, Python 3.8, using a GeForce RTX 3090 GPU for training. With grid search method for tuning hyperparameters, we use Adam as the training optimizer with 120 iterations, an initial learning rate of $1\times {10}^{-5}$, 50% decay every 30 iterations, and a Batch size of 16. The number of feedforward network layers L is 5, and the three fully-connected layers in FC Layers have output dimensions of 4096, 2048, and 2. In addition, we divide the ASD data in Section“Materials and methods” randomly into a training set and a test set in the ratio of 8:2 ratio randomly into training set and test set for subsequent experiments.

### Evaluation metrics

We used Accuracy, Sensitivity and Specificity as metrics to evaluate the ASD identification results. All methods are tested using these metrics and calculated as follows (16–18):

16 $${\text{Accuracy}}=\frac{\mathrm{TruePostive }+\mathrm{ TrueNegative}}{\mathrm{TruePostive }+\mathrm{ FalsePostive }+\mathrm{ TrueNegative }+\mathrm{ FalseNegative}}$$

17 $${\text{Sensitivity}}=\frac{{\text{TruePostive}}}{\mathrm{TruePostive }+\mathrm{ FalseNegative}}$$

18 $${\text{Specifificity}}=\frac{{\text{TrueNegative}}}{\mathrm{TrueNegative }+\mathrm{ FalsePostive}}$$

where True Positive indicates the number of ASD-positive patients correctly classified, True Negative indicates the number of ASD-false-negative patients, False Positive indicates the number of ASD-false-positive patients, and False Negative indicates the number of ASD-negative patients correctly classified.

### Experimental results and discussion

#### Effects of loss function

**Fig. 4:**
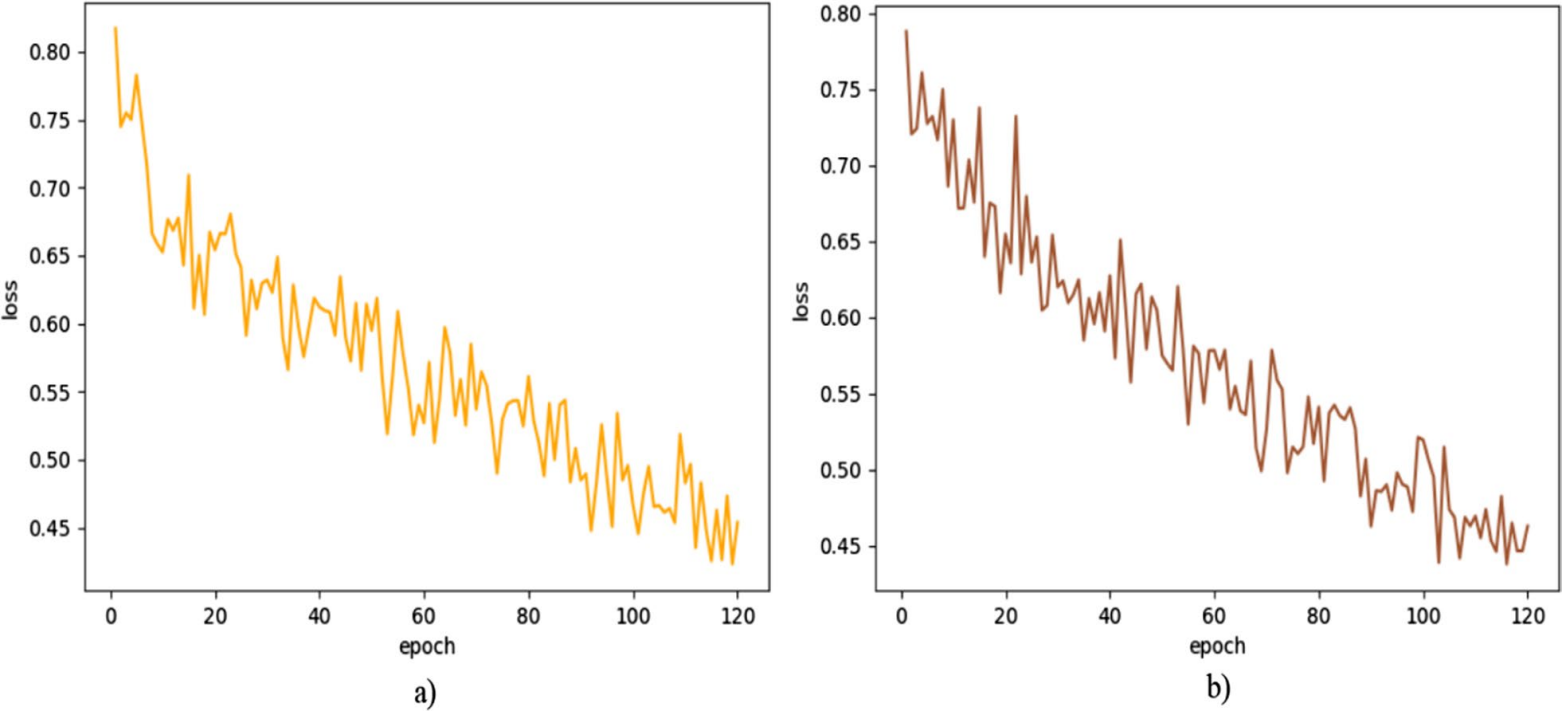
Training loss plots. **a** NYU corresponding loss plot. **b** NYU corresponding loss plot

This experiment established two datasets. Figure 4 shows the loss plot lines during the training of the experiments. For the Fig. 4a and b loss function, the loss value curve has fluctuated several times in a large range during the training process, which may indicate the occurrence of gradient explosion, resulting in excessive weight update of the model, thus causing instability of the model. Therefore, we need multiple training to improve the stability of training. From the graphs, the following conclusions can be drawn: (1) The experiments have converged for both datasets and the experimental results are reliable; (2) The experiments both have the fastest rate of decline until 90 iterations. This indicates that the model was able to effectively learn how to classify ASD patients and NC patients during this time; and (3) The experiments both reached convergence at 105–120 iterations, and the model was able to fit the training data. In summary, our model can fit the ASD dataset well and the experimental results are reliable and valid.

#### Confusion matrix

**Fig. 5:**
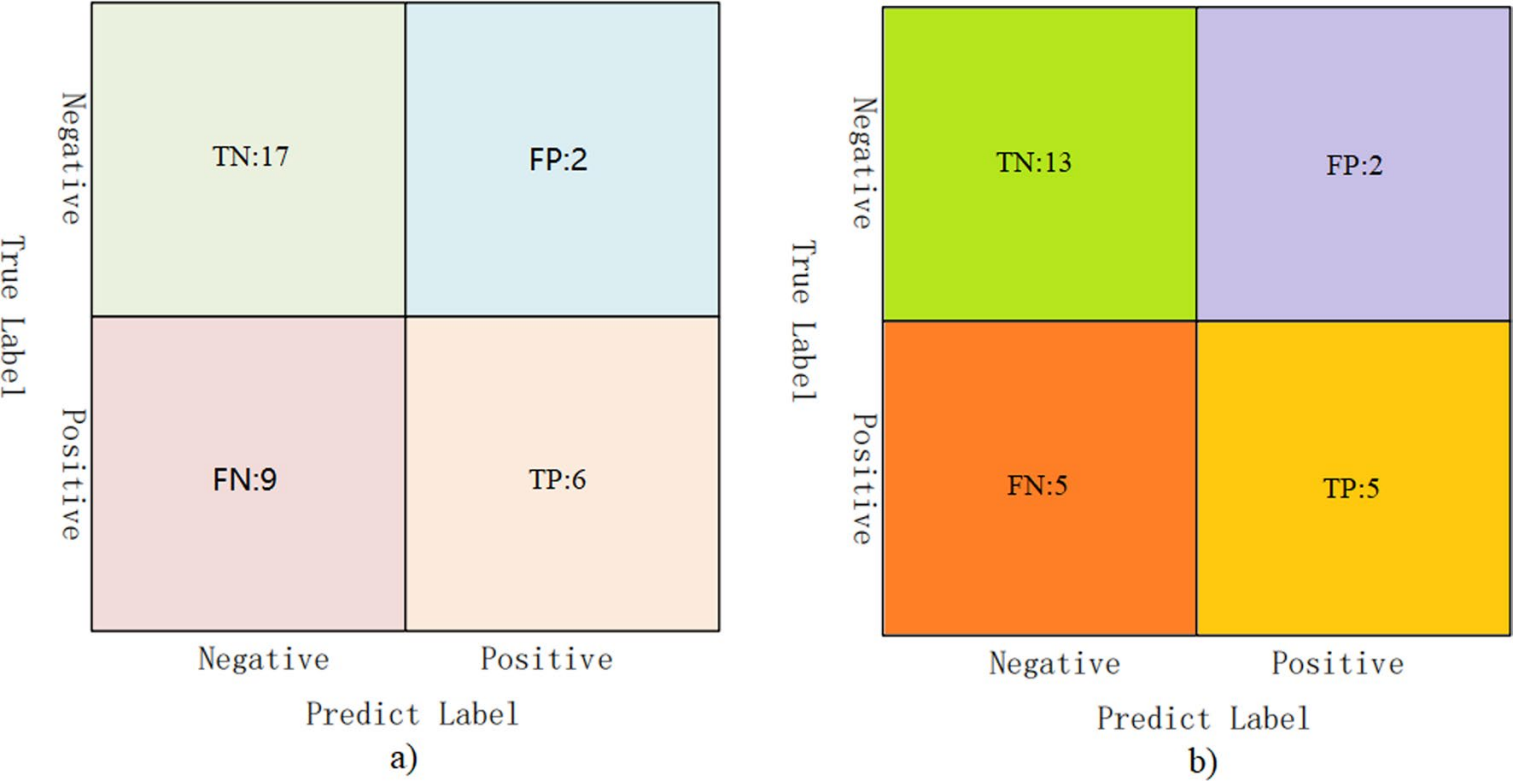
Confusion matrix. **a** Confusion matrix corresponding to NYU dataset. **b** Confusion matrix corresponding to UM dataset

Figure 5 is a confusion matrix showing the number of true negative (TN), false positive (FP), false negative (FN) and true positive (TP) samples. From the figure, we can observe that (1) the TN value is the largest among the four values, i.e., the number of correctly predicted NC samples is the largest. Meanwhile, FP is the smallest, i.e., the number of incorrectly predicted NC samples is the least. This again validates that our method has a low misdiagnosis rate; (2) TP indicates the number of samples that correctly identified ASD patients. The difference between the TP and FN values is not significant. The reason for this result is that the number of NC samples in the training sample is high, which leads to category imbalance and thus affects the ability of the model to identify ASD patients; and (3) In the confusion matrix corresponding to the two datasets, the proportions of TN, FP, FN and TP are similar, which proves that the model has some generalizability. In summary, our method can identify NC patients well and has some ability to identify ASD patients.

### Ablation studies

As shown in Fig. 1, Multitasking transformer framework diagram can be regarded as a federated network composed of multiple features share modules. In this section, we conduct ablation studies to verify the effectiveness of the crucial components in multi-task learning framework and evaluate the impact of each single task network on the results. The transformer network consists of several attention modules to improve the ASD prediction performance. Based on the transformer network, we built a single network and a feature sharing module respectively. All experiments are performed with the same hyperparameter configuration. Table 2 shows the ablation studies with different network configurations.

**Table 2:** Ablation studies with different network configurations

Site	Method	Accuracy (%)	Sensitivity (%)	Specificity (%)
NYU	Single task	63.15	52.63	73.68
	Ours	67.64	40.00	89.47
UM	Single task	70.68	66.00	73.68
	Ours	72.00	50.00	86.66

From index in Table 2, we can see that when we simply add a single task network to the transformer network, Accuracy and Specificity all suffer a decline, but the sensitivity suffers a rise. This shows that adding the multi-task will bring better results. When we applied Single task network and Multitask network to the NYU dataset, the accuracy, sensitivity, and specificity indicators of Single task network were 63.15%, 52.63%, and 73.68%, respectively. The accuracy, sensitivity, and specificity indicators of Multitask network were 67.64%, 40.00%, and 89.47%, respectively. When we applied Single task network and Multitask network to the UM dataset, the accuracy, sensitivity, and specificity indicators of Single task network were 70.68%, 66.00%, and 73.68%, respectively, while the accuracy, sensitivity, and specificity indicators of Multitask network were 72.00%, 55.00%, and 86.66%, respectively. In summary, adding the feature sharing module to the transformer network has the best recognition and prediction performance for rs-fMRI data, indicating the necessity of the feature sharing module in deep learning networks.

### Comparison with the state-of-the-art methods

**Fig. 6:**
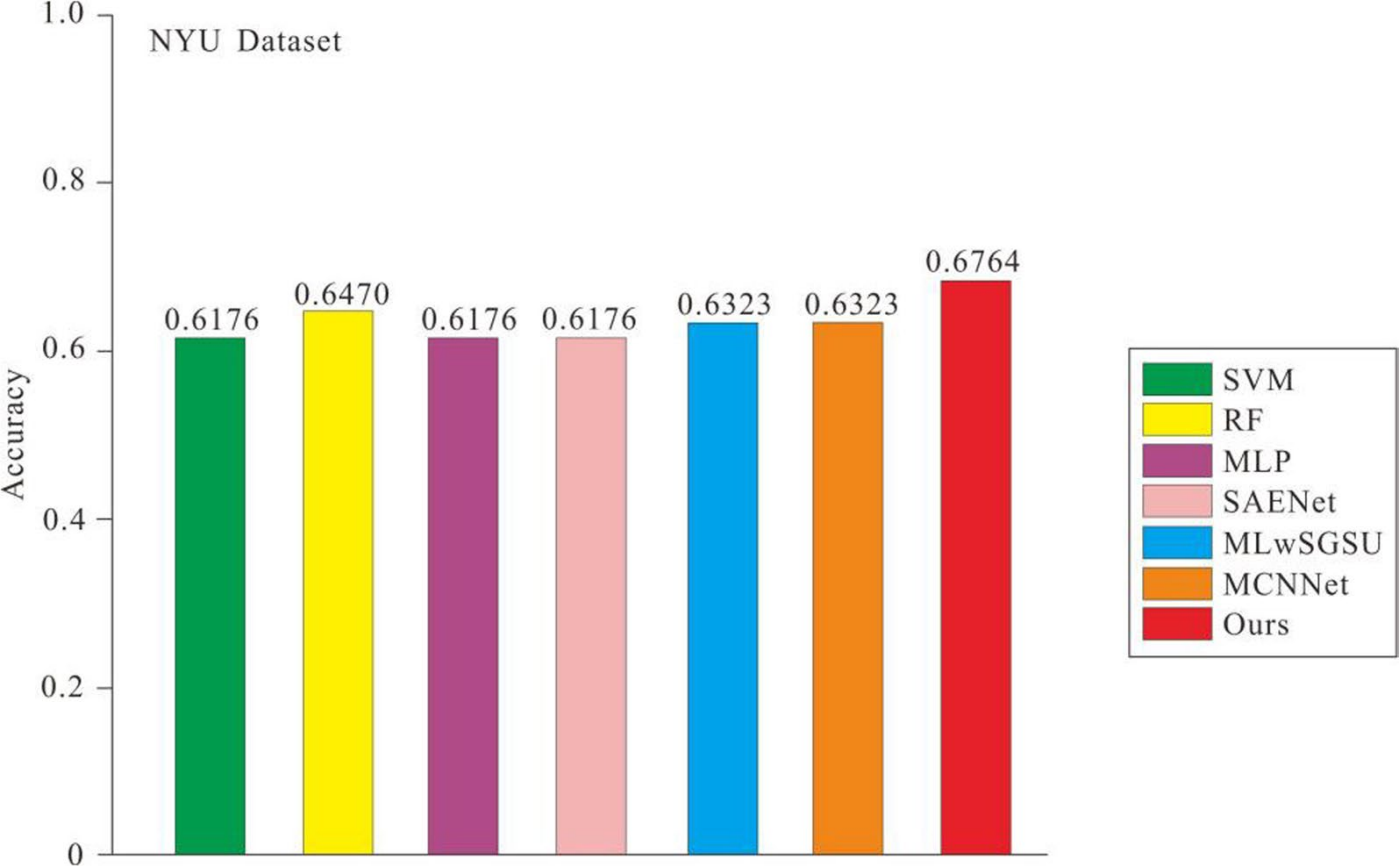
Accuracy comparison of different network models in NYU dataset

**Fig. 7:**
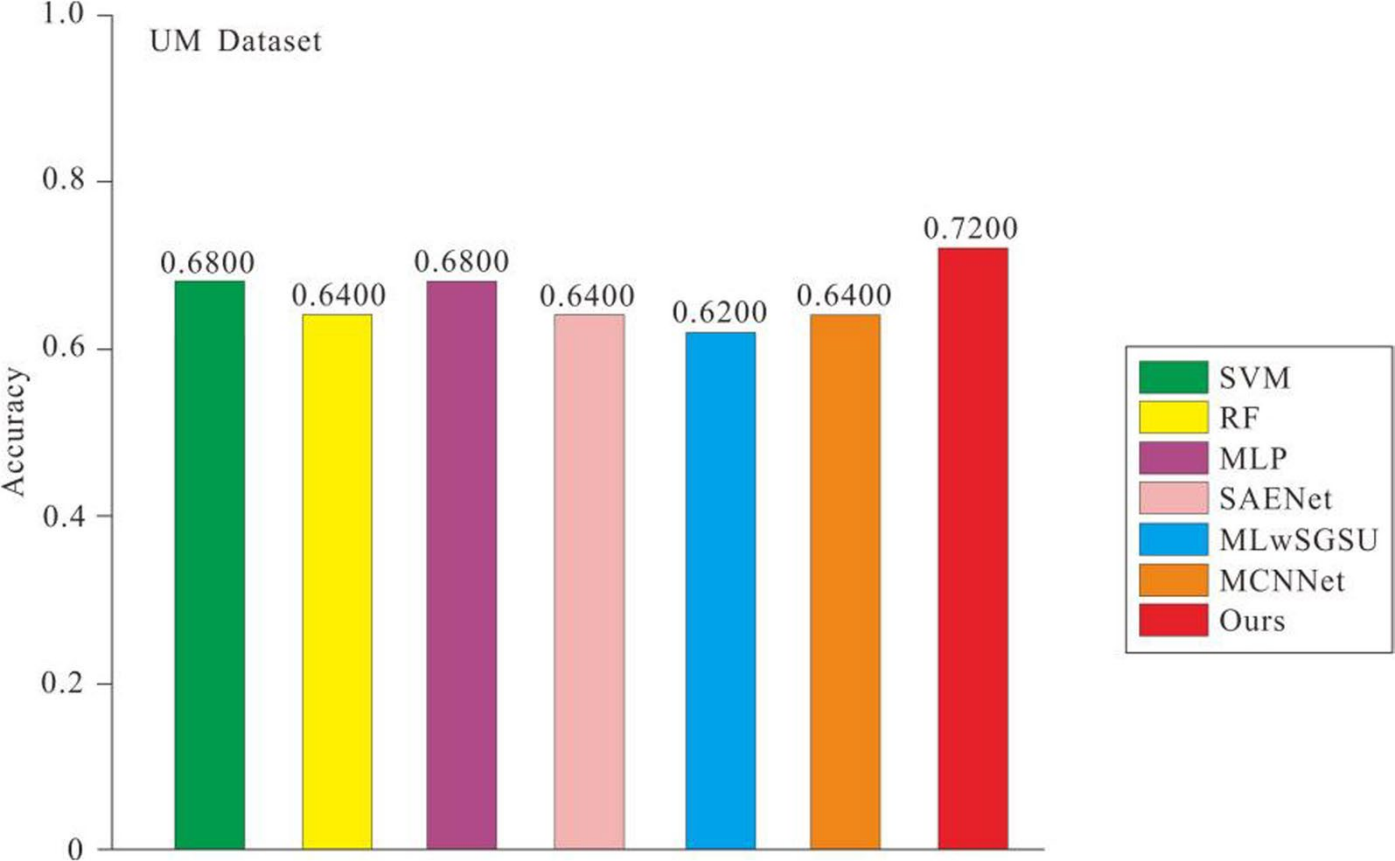
Accuracy comparison of different network models in UM dataset

In this section, we compare the proposed method with some popular machine learning and deep learning methods, including support vector machines [30], random forests [31], multilayer perceptron [32], SAENet [33], MLwSGSU [34] and MCNNet [35]. To test the results of these methods, we used their public codes on the NYU and UM datasets for training and evaluation. The experimental results of the seven models on the two datasets are shown in Figs. 6 and 7. The figures show that compared with other methods, the accuracy values obtained by us have better results. Figures 6 and 7 show that compared with the suboptimal method, the accuracy values obtained by us have increased by 4.54% and 5.88% on the two data sets respectively.

Table 3 shows the results of the different methods on the NYU and UM datasets. On the NYU dataset, our proposed model achieves 67.64%, 40% and 89.47% in accuracy, sensitivity and specificity respectively, which are the best results among all compared methods. Unlike the case on the NYU dataset, on the UM dataset, our proposed model achieves 72%, 50% and 86.66% in accuracy, sensitivity and specificity respectively. Through qualitative comparisons on the two datasets, we find that both our model can guarantee the improvement of comprehensive performance and maintain a high specificity without introducing too many false positives. Therefore, compared to other methods, we believe that Multitasking Transformer framework can better cope with the ASD prediction.

**Table 3:** Experimental results of different methods

Site	Method	Accuracy (%)	Sensitivity (%)	Specificity (%)
NYU	SVM	61.76	46.66	73.68
	RF	64.70	46.66	78.94
	MLP	61.76	46.66	73.68
	SAENet	61.76	40.00	78.94
	MLwSGSU	63.23	53.33	71.05
	MCNNet	63.23	49.99	73.68
	Ours	67.64	40.00	89.47
UM	SVM	68.00	40.00	86.66
	RF	64.00	40.00	80.00
	MLP	68.00	70.00	66.66
	SAENet	64.00	60.00	66.66
	MLwSGSU	62.00	65.00	60.00
	MCNNet	64.00	50.00	73.33
	Ours	72.00	50.00	86.66

To sum up, (1) Multi-task learning methods are competitive with traditional machine learning methods and deep learning methods in ASD recognition; (2) Our methods are significantly better than other methods in both accuracy and specificity; and (3) Our methods are not as sensitive as other methods. We hypothesize that since the number of NC patients in the dataset is slightly more than that of ASD patients, the attention mechanism when training the model is more biased towards learning to capture NC features, thus negatively influencing the extraction of ASD features, and therefore less sensitive. In addition, the method is effective for ASD identification. In conclusion, our method can better identify ASD patients with a lower probability of misdiagnosis of NC patients.

### Limitations

Although our method performs very well compared to other methods, several limitations exist. Although our method has higher accuracy and specificity, there is still lower sensitivity. And the accuracies are only 67.64% and 72%. This is attributed to the amount of training data being too small, which leads too poor generalization on the rs-fMRI data. We plan to explore more effective data augmentation techniques in future work.

## Conclusion

In this study, we propose the multi-task Transformer network, which are essential for predicting and diagnosing ASD diseases. The proposed network utilizes multi-task learning and attention mechanisms for ASD recognition and achieves excellent classification performance on NYU and UM ASD datasets. In addition, the attention mechanism enhances the model's attention to ASD-related features. Multi-task learning enhances the model generalization performance by fusing knowledge learned from different ASD datasets. We evaluated our method on two public datasets and found that it outperformed several state-of-the-art methods with high performance. The results show that combining multitask learning and attention mechanism can better classify ASD patients and NC patients.

## Data Availability

Supplementary material for this article is available on network data set. http://fcon_1000.projects.nitrc.org/indi/abide/.
